# Clinical Implications of Recent Trials on Anticoagulation in Patients with Atrial Fibrillation

**DOI:** 10.5812/cardiovascmed.6541

**Published:** 2012-11-01

**Authors:** Arash Arya, Simon Kircher, Andreas Müssigbrodt, Charlotte Eitel, Philipp Sommer, Gerhard Hindricks

**Affiliations:** 1 Department of Electrophysiology, Heart Centre University of Leipzig, Leipzig, Germany

**Keywords:** Anticoagulation, Artrial Fibrillation

## 1. Introduction

The past year has seen the publication of results of the largest and arguably the most significant clinical trials of antithrombotic to prevent stroke and systemic thromboembolism in patients with atrial fibrillation (AF) (1-3). In 2011, results of two trials concerning anticoagulation in patients with AF in addition to RE-LY study were also published (4-6).

Canadian AF guidelines has been updated early this year, incorporating the new evidence (7). Also in 2012, the American College of Chest Physicians has released guidelines for the antithrombotic therapy of atrial fibrillation (8). Similar updates of ESC and AHA/ACC guidelines are expected, which will be published in the upcoming months. Additionally in 2012, a consensus statement on interventional treatment of atrial fibrillation has been released by HRS/EHRA/ECAS (9). In the present review, new studies will be discussed in the context of current best evidence and their impact on the antithrombotic management of patients with AF will be examined. In addition we present our current clinical practice regarding anticoagulation in patients with AF at Heart Centre University of Leipzig.

## 2. Role of Risk Stratification Scores in the Stroke Prevention

AF is associated with a 10-fold higher mortality rate within 4 months of diagnosis. In the last 2-3 decades, the age and sex-adjusted annual incidence of AF has increased by nearly 13%. Although the incidence of stroke associated with AF has declined in the past decade, probably due to an increase in oral anticoagulant use and better hypertension treatment, the rising incidence and increasing age of the population is predicted to increase stroke burden nearly 80% by 2020 compared to 1990.

**Figure 1.**
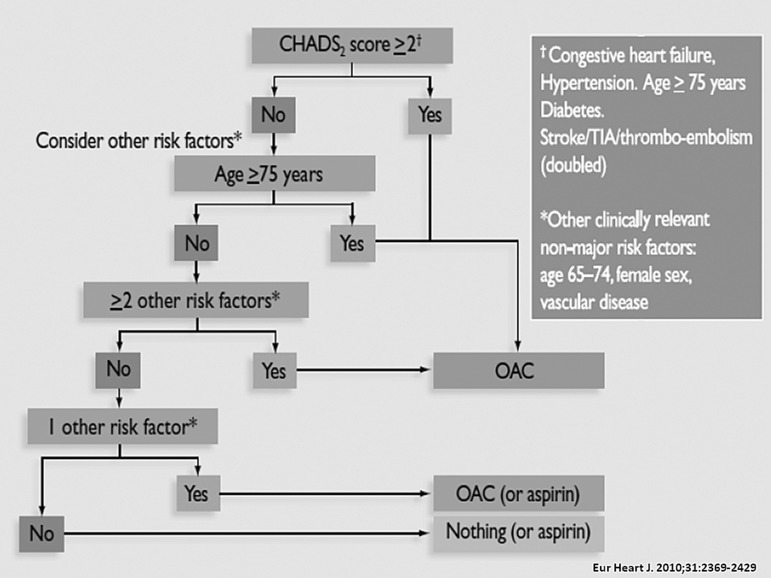
Anticoagulation in Non-valvular Atrial Fibrillation

People over 40 carry a 25% lifetime risk of AF (10). The incidence of strokes attributable to AF increases from 1.5% at the age of 50–59 years to 23.5% at the age of 80–89 years (11). Current ACC/AHA and CCS guidelines recommend the application of the CHADS2 Score, whereas the CHA2DS2-VASc Score is recommended by the European guidelines *(Figure 1) * as a risk stratification tool to assess the risk of stroke and thromboembolism (TE) (1-3, 7). The CHADS2 Score is easier to apply and is better validated than the more recent CHA2DS-VASc Score *(Table 1)*. In contrast to the CHADS2 Score, the CHA2DS2-VASc score allows more precise risk stratification within the population at a low risk for stroke and TE (12-15). Recently published results showed that the CHA2DS2-VASc score gives a more accurate prediction of risks than the CHADS2 score, with risk increasing with each point on the CHA2DS2-VASc scale. Of particular note, the lowest-risk patients on the CHADS2 scale, with a score of 0, were not all actually low risk, with one-year event rates ranging from 0.84 (CHA2DS2-VASc score = 0) to 3.2 (CHA2DS2-VASc score = 3) (13). However, there is a lack of evidence that the CHA2DS2-VASc score is superior to the CHADS2 score regarding clinical choice of antithrombotic therapy and its effect on clinical outcomes.

**Table 1.** Risk Assessment Tools to Predict the Risk of Thromboembolism in Patients with AF.

	CHADS_2_-Score ^a^	CHA_2_DS_2_VASc – Score ^b^
Prior Stroke/TIA ^c^	2	2
Age > 75 years	2	2
Hypertension	1	1
Diabetes Mellitus	1	1
Heart Failure	1	1
Age 64 - 75		1
Vascular Disease		1
Sc ^c^ (Sex cathegory; Female)		1

^a^ Circulation. 2006;114:257-354 (2)

^b^ Eur Heart J. 2010;31:2369-429 (1)

^c^ Abbreviations: AF: Atrial Fibrillation, TIA: Transient Ischemic Attack; Sc: Sex Cathegory

The 2012 CCS Guidelines suggest that patients at low risk of stroke (CHADS2 = 0) should be screened for additional risk factors of stroke (i.e. age 65-74 years, female sex, and presence of vascular disease) (7). Thus, the additional risk factors that are incorporated in the CHA2DS2-VASc Score are taken into account. For patients at low risk (CHADS2-Score = 0) and intermediate risk (CHADS2-Score = 1) the use of additional risk factors will help to improve prediction and probably better prevention of TE events. Since there are a significant number of patients with CHADS2-Score = 0-1 who suffer from TE events, further risk stratification is justified. Retrospective analysis of embolic strokes underlines the advantage of using additional risk factors, like the CHA2DS2-VASc score (12-15). At Leipzig Heart Centre we currently use the CHA2DS2-VASc Score in patients with CHADS2-Score < 2 to further identify patients who probably do not benefit from anticoagulation for prevention of TE events.

## 3. Role of Risk Stratification Scores in the Bleeding Prevention

Due to the existing risk of bleeding, many high-risk patients are not treated with oral anticoagulants. The 2012 CCS guidelines recommended the application of HAS-BLED Score to all patients with AF (7). This score is already included in the 2010 ESC guidelines, mainly to ease the decision-making between the higher and lower dose for Dabigatran (1). However, clinical consequences in case of a high HAS-BLED score are difficult to draw. In addition, an increased HAS-BLED Score always goes along with an increased CHADS2 or CHA2DS2-VASc Score. For most patients the long-term consequences of a stroke outweigh the long-term consequences of bleeding. Moreover, prospective validation of HAS-BLED score is still lacking. The BAFTA Trial has shown that the use of aspirin in elderly patients does not reduce the risk of major bleedings, compared to oral anticoagulants, whereas a significantly higher number of TE events occurred (16). The increased risk of bleeding when multiple anticoagulants are administered is well known and major bleeding is an important contributor to morbidity and mortality in these patients (17). Based on the above mentioned studies acetyl-salicylic-acid currently plays no role for anticoagulation in elderly patients with AF at our centre and it will probably be removed from the guidelines which will be published later this year, according to its high bleeding risk.

## 4. Role of New Oral Anticoagulants in the Stroke Prevention

As recent trials have shown, newer oral anticoagulants offer an improved safety profile, compared to available vitamin-K-antagonists (4-6). The new compounds have more reliable and stable pharmacokinetics, there is no need for monitoring and a fixed dose prescription will provide adequate anticoagulation. The 2011 ACCF/AHA/HRS focused updates suggested that Dabigatran is useful as an alternative to Warfarin to prevent stroke and systemic thromboembolism in patients with paroxysmal to permanent AF and risk factors for stroke or systemic embolization who do not have a prosthetic heart valve, hemodynamically significant valve disease, severe renal failure (GFR < 15 mL/min), or advanced liver disease (impaired baseline clotting function) (3).

The new Canadian guideline suggested that when oral anticoagulation therapy is indicated, most patients should receive Dabigatran (direct thrombin inhibitor) or Rivaroxaban (Factor-Xa inhibitor) instead of Warfarin. This recommendation is based on the above mentioned studies which showed that Dabigatran and Apixaban had greater efficacy and Rivaroxaban had similar efficacy for stroke prevention compared to Warfarin in patients with non valvular AF (4-6). 

Currently, Dabigatran and Rivaroxaban are approved for clinical use and, therefore, in this review we further focus on these two medications *(Table 2-4)*. The preference of one of these new oral anticoagulants over Warfarin is less clear among patients who already receiving Warfarin and have stable anticoagulation (time in therapeutic range > 65%) and no bleeding complications. In addition we have to keep in mind the high financial burden and relatively short clinical experience with these medications. In comparison, there is an extensive clinical experience with warfarin, a specific antidote is available, and we have a simple and standardized test to monitor anticoagulation effect.

**Table 2.** Comparison of Dabigatran and Rivaroxaban, Pharmacokinetics

	DABIGATRAN	RIVAROXABAN
Mechanism of action	Direct Thrombin-Inhibitor	Direct Factor-Xa-Inhibitor
Oral Bioavailability, %	6.5 (3-7) ^a^	80-100
Half-Life, h	12-17	5-13
Renal Elimination, %	80	33
Time to maximum effect, h	1-2	1-4
Potential Interactions	P-Glycoprotein-Inhibitors and inducers	Potent Inhibitors and Inducers of CYP3A4/P-Glycoproteins

^a^ The range of value

**Table 3.** Comparison of Dabigatran and Rivaroxaban: Clinical Studies (1)

	DABIGATRAN	RIVAROXABAN
Study	RE-LY^a^	ROCKET-AF^a^
Number of patients	18113	14264
Dosis	110 mg twice daily^a^ 150 mg twice daily^a^	20 mg daily^a^ 15 mg daily (GFR^a^ < 50 ml/min)
Primary endpoint	Stroke/SE^a^	Stroke/SE^a^
Primary safety issue	Major bleeding	Major Bleeding + MCRB^a^
Study Design	RCT^a^, non-inferiority PROBE, ITT^a^	RCT^a^, Non-inferiority, double blind, double dummy Per-protocol/On treatment Safety-as treated/on treatment
Mean CHADS2-Score	2.1	3.5
CHADS2-Score ≥ 3, %	32.5	87.0
Stroke/TIA^a^ /SE^a^,%	20.0	55
Mean TTR^a^, %	64	55
Typical side effect	Dyspepsia	Epistaxis/Hematuria
NNT^a^	625(110 mg twice daily^a^) 172 (150 mg twice daily^a^)	200

^a^ Abbreviations: AF: atrial fibrillation; GFR: Glomerular Filtration Rate; ITT: Intention to Treat Analysis; MCRB: Minor Clinically Relevant Bleeding; NNT: Number Needed to Treat To Prevent one Primary Endpoint; PROBE: Prospective, Randomized, Open Blinded Adjudication of Events; RCT: Randomized Clinical Trial; SE: Systemic Embolism; TTR: Time in Therapeutic INR Range; TIA: Transient Ischemic Attack; RE-LY: Randomized Evaluation of Long-Term Anticoagulation Therapy; SE: Systemic Thromboembolism

**Table 4.** Efficacy and Safety endpoints in RE-LY and ROCKET-AF Studies

	Dabigatran 110 mg twice daily^a^	Dabigatran 110 mg twice daily^a^	Rivaroxaban
Stroke/SE^**a**^			
Per-Protocol	--	--	Non-Inferior
Safety as treated	--	--	Superior
Intention to treat	Non-Inferior	Superior	Non-Inferior
All Strokes	→^b^	↓	→
Ischemic Strokes	→	↓	→
Hemorrhagic Strokes	↓	↓	↓
Fatal Strokes	→	↓	Trend ↓
Death, Vascular Causes	→	↓	→
All-Cause Mortality	→	Trend ↓	Trend ↓
Major Bleeding	↓	→	→
Intracranial Bleeding	↓	↓	↓
GI^**a**^ Bleeding	→	↑	↑

^a^ Abbreviations: GI: Gastro-Intestinal; SE: Systemic Thromboembolism

^b^ Arrows show the direction of changes (↑ increase, ↓ decrease, → no change)

Initially a questionably increased risk of myocardial infarction in patients who received Dabigatran was suggested, however, recent meta-analysis including the data from the RE-LY trial did not show such an increased risk (7). In addition, in ROCKET-AF (Rivaroxaban vs. Warfarin) and ARISTOTLE (Apixaban vs. Warfarin) studies, the odds ratios for myocardial infarction were 0.81 and 0.61, respectively (5-7). Regular assessment of renal function is of paramount importance in patients who receive new oral anticoagulants as they can accumulate in patients with impaired renal function. Therefore the Canadian Cardiology Society’s Guidelines recommend regular assessment of renal function in patients receiving new oral anticoagulants.

For patients with an estimated glomerular filtration rate (eGFR) > 30 mL per minute, antithrombotic therapy should be used according to their CHADS2 score similar to the patients with normal renal function. The FDA approved dabigatran 150 mg bid for patients with a creatinine clearance greater than 30 mL/min. It also approved dabigatran 75 mg bid for use in patients with a creatinine clearance of 15 to 30 mL/min. However, because of the significant renal excretion of Dabigatran *(Table 2)*, early dosage adaption should be considered early on at an eGFR < 50 ml/min. In such patients Dabigatran 110 mg twice daily or preferably Rivaroxaban 20 mg/day is recommended. Patients with an eGFR 15-30 mL per minute (not on dialysis) should be treated preferably with reduced dose of Rivaroxaban (15 mg/day) or Warfarin. Apixaban has a lower renal excretion (25% renal excretion). Thus, Apixaban (once available for clinical use) might be considered as first-line treatment for patients with chronic renal failure. In Patients with eGFR < 15 mL/min or those who are on dialysis new anticoagulants should *not* be used.

In RE-LY study a total of 1983 cardioversions were performed in 1270 patients: 647, 672, and 664 in the Dabigatran 110 mg, Dabigatran 150 mg, and Warfarin groups, respectively (18). Stroke and systemic embolism rates at 30 days were 0.8%, 0.3%, and 0.6% (Dabigatran 150 mg versus Warfarin, *P* < 0.05) and was similar in patients with and without transesophageal echocardiography. Major bleeding rates were 1.7%, 0.6%, and 0.6% (Dabigatran 150 mg versus Warfarin, *P* = 0.99). These data showed that the frequencies of stroke and major bleeding within 30 days of cardioversion on the 2 doses of Dabigatran were low and comparable to those on Warfarin with or without transesophageal echocardiography guidance. Therefore Dabigatran is a reasonable alternative to Warfarin in patients requiring cardioversion (4, 18). Comparable data are not available for Rivaroxaban, therefore it is not yet recommended as anticoagulation in patients who underwent cardioversion.

Lakkireddy and colleagues studied the role of Dabigatran in patients undergoing catheter ablation of AF (19). In their multicenter (eight high volume centres) observational study, all consecutive patients undergoing AF ablation receiving Dabigatran therapy (with the dose held in the morning of the procedure) were matched by age, sex, and type of AF with an equal number of patients undergoing AF ablation with uninterrupted Warfarin therapy over the same period (19). A total of 290 patients (145 patients in each group), were included in the study. Both groups had a similar CHADS2 score, left atrial size, and left ventricular ejection fraction. Three thromboembolic complications (2.1%) occurred in the dabigatran group compared with none in the Warfarin group (*P* = 0.25). The Dabigatran group had a significantly higher major bleeding rate (6% *vs.* 1%; *P* = 0.019), total bleeding rate (14% *vs.* 6%; *P* = 0.031), and composite of bleeding and thromboembolic complications (16% *vs.* 6%; *P* = 0.009) compared with the Warfarin group. Anticoagulation with Dabigatran was an independent predictor of bleeding or thromboembolic complications (odds ratio: 2.76, 95% confidence interval: 1.22 to 6.25; *P* < 0.01) on multivariate regression analysis. The authors concluded that in patients undergoing AF ablation, periprocedural Dabigatran use significantly increases the risk of bleeding or thromboembolic complications compared with uninterrupted Warfarin therapy (19).

In contrast, winkle and colleagues studied 123 consecutive patients who were started on Dabigatran after catheter ablation of AF. Patients were given enoxaparin 0.5 mg/kg at the end of the procedure, which was repeated 12 hours later and then discontinued. Dabigatran was started 22 hours post-ablation. Primary outcomes were thromboembolic events, bleeding complications, and side effects over a 30-day follow-up period (20). The patients on Dabigatran before ablation (34, 27.6%) with normal renal function had the drug stopped 36 hours *before t*he catheter ablation procedure. There were no pre-procedural or intra-procedural thromboembolic episodes or bleeding. Three patients received Dabigatran 75 mg bid and the rest 150 mg bid. There were no post-ablation strokes, transient ischemic attacks, or systemic thromboemboli in any patient. Three patients discontinued Dabigatran and were switched to Warfarin, 2 because of gastrointestinal side effects and 1 because of a diffuse skin rash. This study showed that Dabigatran is safe and well tolerated after catheter ablation of AF. It did not cause bleeding complications and there were no thromboembolic events. Dabigatran appears to be an alternative to Warfarin after catheter ablation of AF.

**Figure 2.**
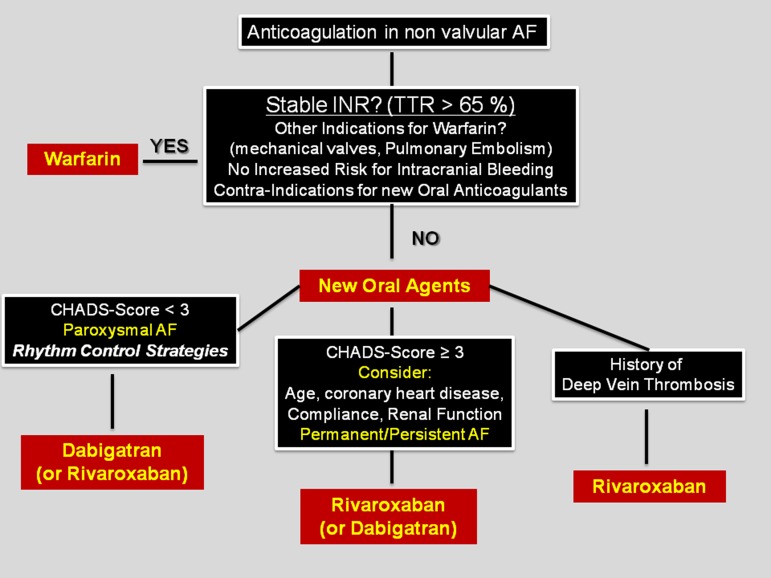
Leipzig Heart Center Algorithm for Anticoagulation in Nonvalvular Atrial Fibrillation

Between July 2010 and September 2011, 89 patients underwent catheter ablation of AF at our centre and received Dabigatran after the procedure. During a mean follow up of 274 days (range 59 – 497) no stroke, transient ischemic attack, or systemic embolism occurred. It seems that Dabigatran, as an anticoagulation after catheter ablation of AF is safe and effective. In patients who are already on Dabigatran, a pause longer than 12 hours might be required. Further studies are needed to clarify this issue. Currently, to the best of our knowledge no data on safety and efficacy of Rivaroxaban after catheter ablation of AF are available. *Tables 3 and 4* show the comparative clinical characteristics of Dabigatran and Rivaroxaban. Based on these data we have already developed a decision algorithm at our centre to choose appropriate oral anticoagulants in patients with AF *(Figure 2)*.

## 5. Role of Catheter Ablation in the Stroke Prevention

Current HRS/EHRA/ECAS guidelines recommend continuing OAC for at least 2-3 months following an AF ablation procedure (9). The decision regarding the continuation of OAC agents more than 3 months following ablation should be based on the patient’s stroke risk factors and not on the presence or type of AF. The discontinuation of systemic anticoagulation therapy post-ablation is not recommended in patients, who are at increased risk of stroke, especially those with prior stroke and/or transient ischemic attack.

Saad and colleagues assessed the long-term outcome of 327 patients after ablation of AF, withholding anticoagulation in patients with sinus rhythm (21). Patients with a CHADS2 score of 2 and 3 accounted for 68.8% of the cohort. After a mean follow-up of 4 years (range, 1.6–8 years), 82% remained free from AF recurrence. All patients were on antiplatelet drugs. No symptomatic ischemic cerebrovascular events were detected despite interruption of anticoagulation in 298 (91%) patients and antiarrhythmic drugs in 293 (89%) patients. Major adverse events were haemorrhagic strokes in 3 patients who continued to receive anticoagulation (21).

These findings should be interpreted cautiously as a recent study showed that CHADS2 score even predicts strokes in patients with coronary heart disease who have no history of AF (22). Therefore, currently the decision regarding anticoagulation after catheter ablation of AF should be based on estimated risk and not the rhythm and/or the outcome of the ablation (23). The CABANA (NCT00911508) and the EAST (NCT01288352) trials are designed to examine the effect of catheter ablation on mortality and morbidity in patients with AF and its role in preventing strokes, cardiovascular deaths, acute coronary syndromes, or decompensated heart failure compared to common therapy (21).

## 6. Cost-effectiveness of New Oral Anticoagulants

Kamel and colleagues recently studied the cost effectiveness of Dabigatran in comparison to Warfarin (24). The authors used a Markov decision model using mainly the data from the RE-LY trial, and the published costs of Dabigatran. They compared the cost and quality-adjusted life expectancy associated with 150 mg Dabigatran twice daily, versus Warfarin therapy targeted to an international normalized ratio of 2–3 . The target population was a cohort of patients aged ≥ 70 years with nonvalvular atrial fibrillation, prior stroke or transient ischemic attack, and no contraindication to anticoagulation. Dabigatran provided 0.36 additional quality-adjusted life-years at a cost of $9000, yielding an incremental cost-effectiveness ratio of $25 000. Dabigatran was cost-effective in 57% of simulations using a threshold of $50 000 per quality-adjusted life-year and 78% of simulations using a threshold of $100 000 per quality-adjusted life-year. The authors concluded that Dabigatran appears to be cost-effective compared to Warfarin for stroke prevention in patients with AF and prior stroke or transient ischemic attack. In sensitivity analyses, as expected, the cost-effectiveness of Dabigatran was inversely related to the quality of international normalized ratio control achieved by Warfarin therapy. Compared to an optimal but uncommon international normalized ratio control (TTR close to 100%) incremental cost-effectiveness ratio would reach $90 000.

## 7. Conclusion

For decades, Warfarin has been the cornerstone of anticoagulation therapy in atrial fibrillation patients. However, it has several limitations including the need for frequent monitoring, inherent variability in response, and propensity for diet and drug interactions. New oral anticoagulants have a consistent and predictable pharmacokinetic profile without significant drug-drug and food-drug interactions and the need for monitoring, thus avoid some of Warfarin’s drawbacks, some related to its “delayed onset of action” and others to difficulties in achieving and maintaining an appropriate degree of anticoagulation.

The RE–LY trial demonstrated that the Dabigatran etexilate is an effective and relatively safe alternative to Warfarin in patients with AF, demonstrating superior efficacy for stroke prevention or systemic embolism (4). It was shown that the Apixaban could be used in patients judged unsuitable for oral anticoagulation with Warfarin. Apixaban has a half-life comparable to Dabigatran and is cleared via multiple elimination pathways suggesting a low potential for clinically important drug interactions (5). Rivaroxaban, studied in the ROCKET-AF trial, showed non-inferiority compared to Warfarin in high risk patients with AF (6). Dabigatran and Rivaroxaban were associated with lower rates of intra-cerebral hemorrhage than Warfarin. Apixaban still awaits approval for clinical use. Generally, the new oral anticoagulants represent a long sought-after advance in medical therapy and are predicted to enable many more patients with AF to receive effective anticoagulant therapy.
